# The deep layer of the tractus iliotibialis and its relevance when using the direct anterior approach in total hip arthroplasty: a cadaver study

**DOI:** 10.1007/s00402-017-2820-x

**Published:** 2017-10-14

**Authors:** David Putzer, Matthias Haselbacher, Romed Hörmann, Günter Klima, Michael Nogler

**Affiliations:** 1 Department of Orthopaedic Surgery, Experimental Orthopaedics, Medical University of Innsbruck, Innrain 36, 6020 Innsbruck, Austria; 2 Department of Orthopaedic Surgery, Medical University of Innsbruck, Anichstrasse 35, 6020 Innsbruck, Austria; 3 Division of Histology and Embryology, Department of Anatomy, Histology and Embryology, Medical University of Innsbruck, Müllerstrasse 59, 6020 Innsbruck, Austria; 4 Division of Clinical and Functional Anatomy, Department of Anatomy, Histology and Embryology, Medical University of Innsbruck, Müllerstrasse 59, 6020 Innsbruck, Austria

**Keywords:** Iliotibial tract, Minimally invasive approach, Total hip arthroplasty, Direct anterior approach, Iliotibial band, Tensor fasciae latae

## Abstract

**Introduction:**

Surgical approaches through smaller incisions reveal less of the underlying anatomy, and therefore, detailed knowledge of the local anatomy and its variations is important in minimally invasive surgery. The aim of this study was to determine the location, extension, and histomorphology of the deep layer of the iliotibial band during minimally invasive hip surgery using the direct anterior approach (DAA).

**Materials and methods:**

The morphology of the iliotibial tract was determined in this cadaver study on 40 hips with reference to the anterior superior iliac spine and the tibia. The deep layer of the tractus iliotibialis was exposed up to the hip-joint capsule and length and width measurements taken. Sections of the profound iliotibial tract were removed from the hips and the thickness of the sections was determined microscopically after staining.

**Results:**

The superficial tractus iliotibialis had a length of 50.1 (SD 3.8) cm, while tensor fasciae latae total length was 18 (SD 2) cm [unattached 15 (SD 2.5) cm]. Length and width of the deep layer of the tractus iliotibialis were 10.4 (SD 1.3) × 3.3 (SD 0.6) cm. The deep iliotibial band always extended from the distal part of the tensor fascia latae (TFL) muscle to the lateral part of the hip capsule (mean maximum thickness 584 μm). Tractus iliotibialis deep layer morphology did not correlate to other measurements taken (body length, thigh length, and TFL length).

**Conclusions:**

The length of the deep layer is dependent on the TFL, since the profound part of the iliotibial band reaches from the TFL to the hip-joint capsule. The deep layer covers the hip-joint capsule, rectus, and lateral vastus muscles in the DAA interval. To access the precapsular fat pad and the hip-joint capsule, the deep layer has to be split in all approaches that use the direct anterior interval.

## Introduction

Our interest in the anatomy of the tractus iliotibialis stems from our focus on minimally invasive total hip arthroplasty (THA). Since surgical approaches through smaller incisions reveal less of the underlying anatomy and, therefore, impair identification of underlying structures, detailed knowledge of the local anatomy and its variations is important in minimally invasive surgery.

The tractus iliotibialis has a very distinct anatomy at the level of the hip joint. The tractus iliotibialis, known also as the iliotibial band, or the band of Maissiat, is a ligament which originates from the iliac crest and extends into the lateral side of the tibia [1]. It is connected intimately with the tensor fasciae latae (TFL) anteriorly and the glutaeus maximus (GM) posteriorly in the region below the greater trochanter (GT) [1–3]. The tractus iliotibialis is not fixed at the GT [4, 5], but uses it as a diversion point [3]. The tendon of the gluteus maximus and a major portion of the tractus iliotibialis intermingle near the gluteal tuberosity.

At the proximal end, the tractus iliotibialis splits into superficial and deep layers, enclosing TFL and anchoring at the iliac crest [3, 6, 7]. A tough layer goes deep and with the iliofemoral ligament to the upper acetabulum [8, 9]. The majority of the vertical bundles of fibrous tissue can be followed up to the iliac tubercle (IT), while part of it attaches to the most lateral lip of the iliac crest [8]. Sher et al. demonstrated that the fascia lata has an insertion along the entire lower border of the iliac crest and the tractus iliotibialis is inserted onto Gerdy’s tubercle distally [2, 3, 10]. Adduction at the hip is limited by the tractus iliotibialis, while extension is limited by the iliofemoral ligament and the deep layer of the tractus iliotibialis [8].

The tractus iliotibialis has to be split in all lateral approaches to the hip. With the direct anterior approach (DAA), however, access is from anterior and stays medial to the anterior border of the tractus iliotibialis [11]. The superficial layer of the tractus iliotibialis, therefore, remains untouched, but its deep layer forms a much stronger band. This band inserts into the fascia of the rectus femoris (RF) muscle and the hip-joint capsule and its deep layer has to be dissected.

**Fig. 1:**
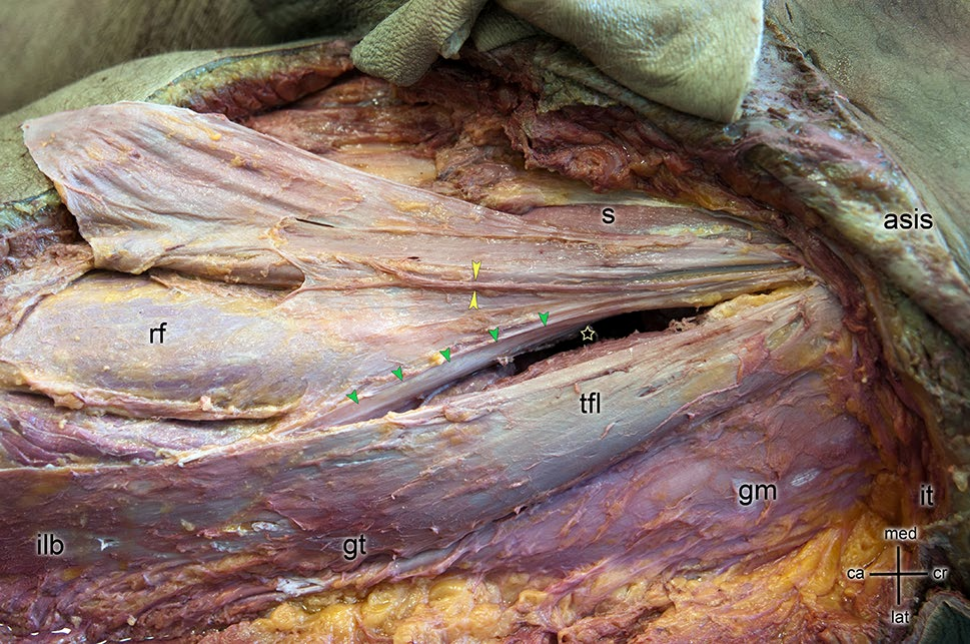
Skin incision is placed lateral and distal to the anterior superior iliac spine (asis) to access the hip through the direct anterior approach (star). The iliac tubercle (it) is shown as a reference point. Skin and subcutaneous fat tissue were removed from the leg exposing the iliotibial band (ilb) and sartorius muscle (s). Special care was taken to keep the ilb and the tensor fasciae latae muscle (tfl) intact. After dissecting the interval between the tensor fasciae latae muscle (tfl), sartorius muscle (s), and rectus femoris muscle (rf), a band of strong fibers extending from proximal lateral to distal medial becomes visible (green arrows). Typically, this structure covers the anterior precapsular fat pad and connects the superficial layer of the ilb with the fascia of the rf and the fascia of the lateral vastus muscle. This anatomical structure is the deep layer of the ilb which needs to be split or resected to reach the hip-joint capsule using a direct anterior approach. With yellow arrows, the nervus cutaneous femoris lateralis is indicated. For orientation, the great trochanter (gt) and musculus gluteus medius (gm) are indicated

Using the DAA, the skin incision is usually placed lateral and distal to the anterior superior iliac spine. The subcutaneous fascia is exposed and split. After dissecting the interval between the TFL, sartorius muscle (SM), and rectus femoris muscle (RF), a band of strong fibers extending from proximal lateral to distal medial becomes visible (Fig. 1).

Typically, this structure covers the anterior precapsular fat pad and connects the superficial layer of the tractus iliotibialis with the fascia of the RF and the fascia of the lateral vastus muscle (LV). This anatomical structure is the deep layer of the tractus iliotibialis which needs to be split or resected to reach the hip-joint capsule. In our experience, this part of the band varies greatly in strength and thickness.

Divergent descriptions of the anatomic locations and biomechanical function of tractus iliotibialis can be found in the literature [2, 3] and little information is available on the deep layer of the tractus iliotibialis in the anatomy literature [12, 13]. In some publications by Benninghoff et al. [5], Henle et al. [14], Lanz and Wachsmuth [15], Platzer [16], Schünke et al. [17], Sieglbauer et al. [18] and Waldeyer and Waldeyer [19], no reference to the hip-joint contact or indeed the deep layer itself was found.

To reach the hip-joint capsule in all lateral, anterolateral, and anterior approaches, this deep layer has to be cut or split. Therefore, to provide essential information to the orthopedic surgeon, information on the anatomy and morphology of the deep layer of the tractus iliotibialis is needed.

The aim of this study was to determine the location, extension, and histomorphology of the deep layer of the tractus iliotibialis band to provide more thorough information to the surgeon during minimally invasive hip surgery using the DAA.

## Materials and methods

Twenty formaldehyde fixated human cadavers were used in this study. All cadavers were donated to the Division of Functional Anatomy of the Medical University Innsbruck for research. Informed consent was obtained from all individual participants included in the study. Both hips of 20 specimens were included, resulting in a number of 40 measurements.

### Specimen preparation

The skin and subcutaneous fat tissue were removed from the leg and pelvis and the superficial muscular fascia was prepared. Special care was taken to keep the tractus iliotibialis and the TFL intact. After preparation, the fascia at the anterior border of the TFL was split, the interval between TFL and S and RF was opened, and the deep layer of the tractus iliotibialis protected (Fig. 1). The fat tissue around the deep layer was removed until the entire deep layer was visible.

### Measurements

All measurements were made under full visualization by two of the authors together (MH, MN). Table 1 shows the variables measured. All variables except body mass index (BMI) were measured in mm.

**Table 1:** Quantitative measurement of the anatomical structures

	Mean	Range
Age (years)	76	55–88
Body length (mm)	1688	1520–1903
BMI (kg/m^2^)	28	18–36
Leg length (mm)	923	810–1101
Thigh length (mm)	494	428–600
Length of the superficial tractus iliotibialis (mm)	501	431–605
Width deep layer of the tractus iliotibialis (mm)	33	24–49
Length deep layer of the tractus iliotibialis (mm)	104	75–140
TFL total length (mm)	178	142–224
Unattached TFL length (mm)	149	50–192
Thickness of histological slices (μm)	584	155–1374

**Fig. 2:**
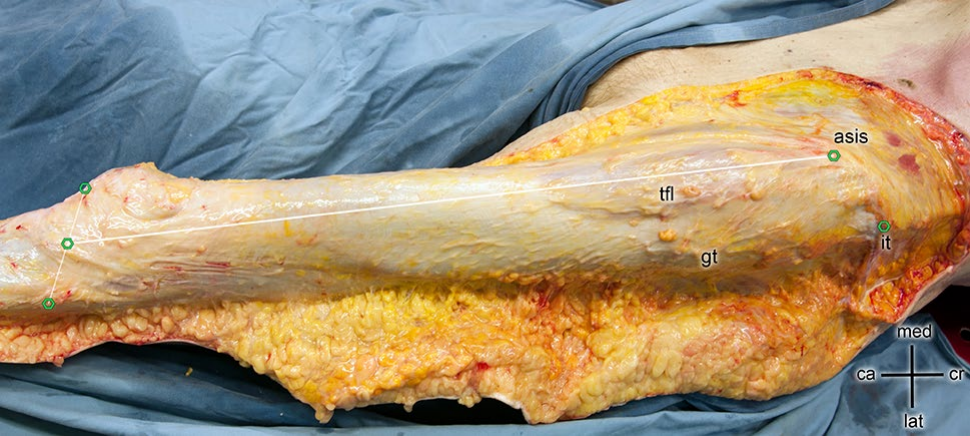
The length of the superficial layer of the tractus iliotibialis was determined by setting up a straight line from the anterior superior iliac spine (asis) to the tibia. The endpoint at the tibia was identified by setting up a straight line between the caput fibulae and the apex patellae at the height of the tibia plateau (green points)

The length of the superficial layer of the tractus iliotibialis was determined by setting up a straight line from the anterior superior iliac spine (ASIS) to the tibia. The endpoint at the tibia was identified by setting up a straight line between the caput fibulae and the apex patellae at the height of the tibia plateau (Fig. 2).

The fascia was cut along the line between the ASIS and the tibia plateau to expose the TFL. Platzer [16] revealed that the origin of the TFL is in the area of the ASIS. The deep layer of the tractus iliotibialis is located medial to the TFL. It was bluntly dissected up to the point, where it split from the TFL. The proximal part of the TFL not attached to the deep layer was measured by measuring the TFL from the ASIS to the split-off point. The width of the deep layer of the tractus iliotibialis was measured 30 mm proximal from the split-off point of the tractus iliotibialis.

**Fig. 3:**
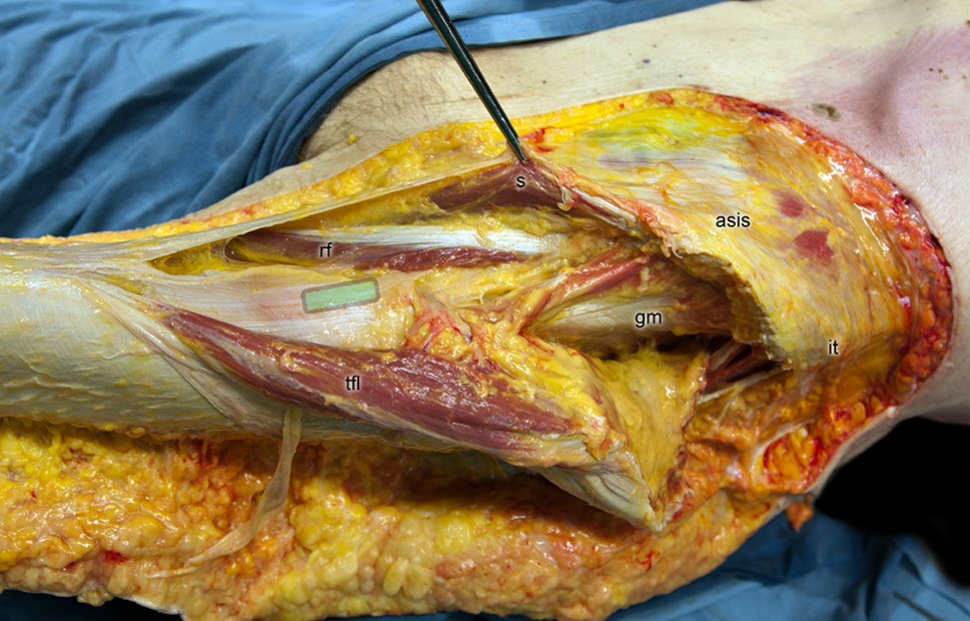
The 20 mm by 10 mm sample for histology of the deep layer of the tractus iliotibialis was obtained proximal to the split-off point in the middle of the proximal–distal extension (magnified cross-section green area). The slice was taken parallel to the fibers of the deep layer

The deep layer of the tractus iliotibialis was exposed up to the hip-joint capsule. A probe was advanced from the insertion of the deep layer into the hip-joint capsule to the point, where the layer splits off from the TFL to measure the length of the profound tractus iliotibialis. The deep layer was removed after these measurements. A first cut was made at the junction of the upper and deep layer. A second cut was performed at the insertion of the deep layer into the fascia of LV and RF. The orientations of the specimens were marked (anterior–posterior, proximal–distal) on the specimens. A 20 mm by 10 mm sample for histology of the deep layer of the tractus iliotibialis was obtained proximal to the split-off point in the middle of the proximal–distal extension. The slice was taken parallel to the fibers of the deep layer (Fig. 3).

### Preparation of the histological sample

After postfixation in 10% formalin, the specimens were washed for 2 h under running water and then totally dehydrated in ascending concentrations of isopropanol alcohol (70, 80, 90% and 2 × 100%) according to a study by Junqueira et al. [20].

Subsequently, for 12 h each, the specimens were soaked for 24 h in methyl benzoate which blends with the dehydrator and with paraffin as revealed by Bucher et al. [21].

The specimens were exposed to chloroform for 20 min to enhance the infiltration of the liquid Paraplast^®^ (Structure Probe Inc.) into the material. During the next step, the specimens were submerged in liquid Paraplast for 2–3 days for total infiltration. The material was then poured into a cubic block. During dehydration, the material is subject to change due to the loss of water, the infiltration of liposoluble agents, and increased temperature. Shrinkage of up to 40 volume percent (vol.%) is possible, depending on the fixative as revealed by Bucher et al. [21].

The fixed specimens were sectioned into slices of 7–10 µm with a sliding microtome (Leitz 1400, Leica Microsystems, Nussloch, Germany). The material was then stained on the stage. The most common staining for a general overview is hematoxylin and eosin staining (HE) which colors the cell nuclei, the cytoplasm, and the collagen fibers. This method revealed by Bucher et al. [21] is gradual, since the material is first exposed to hematoxylin and then to eosin, and results in differentiation (i.e., intensification or reduction of the individual staining).

**Fig. 4:**
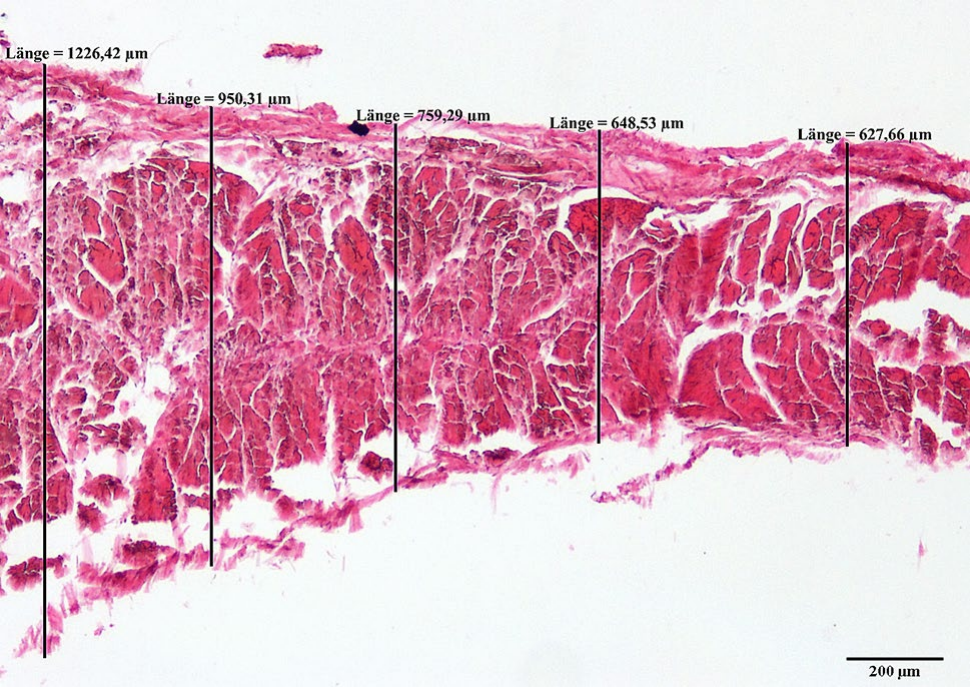
Example of a histological slice of the deep layer of the tractus iliotibialis for measuring the thickness

The cross-sectional thickness of the material was photographed with a Nikon Eclipse (Eclipse 80 I, Nikon, Tokyo, Japan) and the pictures were processed with the software NIS Elements (Version BR 3.00, SP7, Nikon, Tokyo, Japan) at different points with a spacing of 200–500 µm. For further processing, the maximum thickness of four measurements was calculated (Fig. 4).

Exact Kolmogorov–Smirnov tests were performed to test for normal distribution of all variables. Dependent sample *t* tests were chosen to test for significant differences between left and right hips. The ratios between the anatomical structures (thigh length, body height, leg length, length of the superficial tractus iliotibialis, and the length of the deep layer of the tractus iliotibialis) were calculated. Two-tailed probability values less than 0.05 were considered significant. Spearman rank correlations were calculated for correlation analysis. Statistical analysis was performed using SPSS (Version 17.0, SPSS Inc, Chicago, IL, USA).

## Results

The deep layer of the tractus iliotibialis had shrunk by 30% during preparation due to the histological processing.

Table 1 shows the main results regarding the anatomical structures measured, including the histological slices. Leg length was around 92 (SD 6.7) cm and tight length around 50 (SD 4) cm. Length and width of the deep layer of the tractus iliotibialis were 10.4 (SD 1.3) × 3.3 (SD 0.6) cm. The superficial tractus iliotibialis had a length of 50.1 (SD 3.8) cm, while TFL total length was 18 (SD 2) cm [unattached 15 (SD 2.5) cm].

TFL was laying between the superficial and the deep layer of the tractus iliotibialis. We found that 83% (25–99) of the TFL muscles were covered but not connected to the deep layer of the tractus iliotibialis.

Table 2 shows the ratios between measured anatomical structures. A mean of 99% for tight length to superficial tractus iliotibialis was found. The mean ratio between deep layers of the tractus iliotibialis to thigh length was 21%.

**Table 2:** Ratios between the anatomical structures

	Mean (%)	Range (%)
Thigh length to body length	29	27–32
Thigh length to leg length	54	51–58
Thigh length to superficial tractus iliotibialis	99	94–113
Deep layer of the tractus iliotibialis to thigh length	21	14–30

*P* values for the correlations between body length, thigh length, TFL length, and Tractus iliotibialis deep layer length, width, and thickness are listed in Table 3. A statistical significant correlation could be found between the superficial tractus iliotibialis length and body length, leg length, tight length, and FL length. The correlation between Tractus iliotibialis deep layer length and width as well as the thickness did not correlate with any of the other measurements taken (body height, thigh length, and TFL length) except with the BMI (*P* = 0.014).

**Table 3:** Correlations between the anatomical structures

	Level of Significance				
	BMI	Body length	Leg length	Thigh length	TFL length
Body length	< 0.001	–	< 0.001	< 0.001	0.001
Leg length	< 0.001	< 0.001	–	< 0.001	< 0.001
Thigh length	0.006	< 0.001	< 0.001	–	< 0.001
TFL length	0.003	0.001	< 0.001	< 0.001	–
Superficial tractus iliotibialis length	0.012	< 0.001	< 0.001	< 0.001	0.001
Tractus iliotibialis deep layer length	0.655	0.164	0.124	0.129	0.024
Tractus iliotibialis deep layer width	0.306	0.284	0.227	0.529	0.026
Tractus iliotibialis deep layer thickness	0.014	0.236	0.273	0.764	0.886

## Discussion

One risk of the DAA is damage to the area of the lateral femoral cutaneous nerve (LFCN) with or without formation of scar tissue (Fig. 1, yellow arrows) [22, 23]. The standard strategy to avoid this is to split the TFL fascia laterally and to continue preparation strictly subfascially. The deep layer of the tractus iliotibialis is seen as a thick band that covers the hip-joint capsule and connects the fasciae of the rectus muscle and lateral vastus muscle.

The tractus iliotibialis consists of three layers: the superficial layer, intermediate layer, and deep layer [9, 24, 25]. These layers fuse in the region of the greater trochanter and form the proximal tractus iliotibialis [3, 24]. The superficial layer arises from the ilium superficial to the TFL, while the intermediate layer arises from the ilium slightly below the origin of the TFL and lies deep to the muscle [24, 25]. The superficial and intermediate layers of the tractus iliotibialis merge at the distal end of the TFL and serve as the tendon for the TFL [3, 24]. The deep layer is described by Huang et al. as a constant structure arising from the supraacetabular fossa between the hip capsule and the tendon of the reflected head of the rectus femoris [24, 25]. This deep layer merges just distal to where the superficial and intermediate layers fuse [24, 25]. In a direct anterior approach with an incision length ranging from 60 to 100 mm according to Bender et al. [26], this structure extends throughout the surgical field, and was also found in our cadavers.

The length of the superficial tractus iliotibialis correlated with the length of the thigh and the length of the body. Fanghänel et al. [27] revealed that the main reasons for these correlations are the bony point of origin (ASIS) and the insertion of the tractus iliotibialis (tuberculum tractus iliotibialis).

Thigh length and body length did not correlate significantly with the length of the deep layer. A weak correlation was found between the length of the TFL and the length of the deep layer (*r* = 0.353). The length of the deep layer is dependent on the TFL, since the profound part of the tractus iliotibialis reaches from the TFL to the hip-joint capsule.

We demonstrated that a deep layer of the tractus iliotibialis exists and covers the hip-joint capsule, rectus, and lateral vastus muscles in the DAA interval. It extends from the origin of the TFL and inserts into the tractus iliotibialis together with the insertion of the TFL into the tract. The mean length of the deep layer was 10 cm and its length depends on that of the TFL. Therefore, it can be seen as the inner layer and as an envelope covering the TFL. To gain access to the precapsular fat pad and the hip-joint capsule, this deep layer has to be split in all approaches that use the DAA interval for THA.
